# Phenotypic and Genotypic Evaluation of Carbapenemases in Carbapenem-Resistant *Klebsiella pneumoniae* Isolates and Investigation of Ceftazidime-Avibactam and Colistin Susceptibility

**DOI:** 10.3390/ijms27209307

**Published:** 2026-10-20

**Authors:** Elif Gülsüm Torun, Elif Okumuş, Esra Kaya

**Affiliations:** 1 Department of Medical Microbiology, Kahramanmaraş Necip Fazıl City Hospital, 46000 Kahramanmaraş, Türkiye; esra_ytn@hotmail.com; 2 Department of Microbiology, University of Health Sciences Van Training and Research Hospital, 65300 Van, Türkiye; drelifokumus@gmail.com

**Keywords:** *Klebsiella pneumoniae*, carbapenem resistance, NDM, OXA-48, mCIM, eCIM, ceftazidime-avibactam, colistin

## Abstract

Carbapenem-resistant *Klebsiella pneumoniae* (CR-Kp) is a major healthcare-associated pathogen, and the coexistence of different carbapenemases may complicate phenotypic detection and antimicrobial treatment. This study evaluated carbapenemase profiles in 123 CR-Kp isolates from two centers in Türkiye and compared the modified carbapenem inactivation method (mCIM) and EDTA-modified carbapenem inactivation method (eCIM) with multiplex real-time PCR targeting KPC, NDM, VIM, IMP, and OXA-48. Ceftazidime-avibactam (CZA) and colistin susceptibility were also assessed. Carbapenemase genes were detected in 122/123 isolates (99.2%), predominantly NDM + OXA-48 (45.5%), followed by OXA-48 alone (26.8%), KPC alone (18.7%), and NDM alone (8.1%). mCIM showed 99.2% sensitivity, whereas eCIM showed 87.7% sensitivity and 100% specificity for NDM detection. All eight false-negative eCIM results occurred among NDM + OXA-48 co-producing isolates (8/55), whereas no false-negative results were observed among isolates carrying NDM alone (0/10); this difference was not statistically significant (*p* = 0.339). CZA resistance was observed in 49.6% of isolates, with a higher likelihood of resistance among NDM-positive isolates in the exploratory analysis (OR, 9.03; 95% CI, 3.97–20.54; *p* < 0.001). Colistin resistance was observed in 36.6% of isolates, with no statistically significant association with carbapenemase profiles in the analyses performed. These exploratory findings suggest that combined molecular and phenotypic testing may be useful for carbapenemase characterization and highlight the occurrence of false-negative eCIM results in some NDM + OXA-48 co-producing isolates. Given the absence of adjustment for multiple comparisons, the observed associations should be interpreted cautiously and considered hypothesis-generating.

## 1. Introduction

Carbapenem-resistant *Klebsiella pneumoniae* (CR-Kp) has become an important public health concern worldwide due to its increasing prevalence, potential for spread in healthcare settings, and limited treatment options. CR-Kp is an important cause of healthcare-associated infections, particularly in intensive care units, and is associated with prolonged hospital stays, increased healthcare costs, treatment failure, and increased mortality [1,2]. Because of its global importance, CR-Kp was included among the critical-priority pathogens in the World Health Organization (WHO) Bacterial Priority Pathogens List 2024, highlighting the need for the development of new antimicrobials, strengthened resistance surveillance, and effective infection control strategies [3].

Carbapenemase production is one of the major mechanisms of carbapenem resistance in *K. pneumoniae*. Carbapenemases constitute a heterogeneous group of enzymes capable of hydrolyzing β-lactam antibiotics, including carbapenems. According to the Ambler classification, clinically important carbapenemases include *Klebsiella pneumoniae* carbapenemase (KPC) in class A; New Delhi metallo-β-lactamase (NDM), Verona integron-encoded metallo-β-lactamase (VIM), and imipenemase (IMP) among class B metallo-β-lactamases (MBLs); and OXA-48-like carbapenemases in class D. Class A and D carbapenemases act through a serine-dependent hydrolytic mechanism, whereas class B MBLs require zinc for their enzymatic activity [4].

Although the distribution of carbapenemases varies geographically, KPC, NDM, and OXA-48-like enzymes are among the most clinically important carbapenemase types in *K. pneumoniae*. OXA-48 was first identified in a *K. pneumoniae* isolate from Türkiye and subsequently spread to different geographical regions. OXA-48-like carbapenemases are particularly prevalent in Europe, the Middle East, and North Africa and may coexist with MBLs such as NDM [5]. In recent years, the increasing detection of NDM and isolates carrying more than one carbapenemase gene has further complicated the epidemiology of carbapenem resistance [6,7]. The accurate and timely detection of carbapenemases is important for identifying resistance mechanisms, implementing infection control measures, and epidemiological surveillance. Molecular methods allow for the direct detection of clinically important carbapenemase genes. However, their routine use may not be feasible in all laboratories because of cost, technical infrastructure, and trained personnel requirements. Therefore, phenotypic methods such as the modified carbapenem inactivation method (mCIM) and EDTA-modified carbapenem inactivation method (eCIM) remain useful because they are inexpensive and can be performed under routine laboratory conditions [8,9]. Nevertheless, the performance of phenotypic methods may vary according to carbapenemase type. In particular, the variable performance of eCIM in detecting some MBL types makes it important to interpret phenotypic findings together with molecular results [10].

Identification of the carbapenemase type is important not only for epidemiological purposes but also for evaluating potential antimicrobial treatment options. Ceftazidime-avibactam (CZA) has become an important treatment option for CR-Kp infections. Avibactam inhibits class A carbapenemases such as KPC and class D OXA-48-like enzymes but does not inhibit class B MBLs such as NDM, VIM, and IMP. Therefore, CZA susceptibility is closely related to the carbapenemase genotype of the isolate. Knowledge of the genotypic profile becomes particularly important when more than one carbapenemase gene is present in the same isolate [11,12].

Colistin remains one of the treatment options for CR-Kp infections. However, the increasing prevalence of colistin resistance has become an important factor limiting its effectiveness [13]. In *K. pneumoniae*, colistin resistance may arise through different molecular mechanisms, including chromosomal mechanisms leading to alterations in lipopolysaccharide structure and transferable resistance mechanisms [14]. Because the coexistence of carbapenem and colistin resistance in the same isolate may further restrict treatment options, monitoring colistin susceptibility in CR-Kp isolates remains clinically relevant [13].

In this study, we aimed to evaluate carbapenemases in carbapenem-resistant *K. pneumoniae* isolates using phenotypic and genotypic methods and to assess the agreement between these methods. We also investigated ceftazidime-avibactam and colistin susceptibility and their associations with carbapenemase genotypes. The study was designed to contribute to current data on the molecular epidemiology of carbapenem-resistant *K. pneumoniae* and to characterize the relationships among phenotypic findings, genotypic profiles, and antimicrobial susceptibility results.

## 2. Results

### 2.1. Characteristics of the Study Isolates

A total of 123 carbapenem-resistant *Klebsiella pneumoniae* isolates were included in the study. Of these, 72 (58.5%) were obtained from University of Health Sciences Van Training and Research Hospital and 51 (41.5%) from Kahramanmaraş Necip Fazıl City Hospital. The median age of the patients was 70 years (interquartile range [IQR], 57.5–78), with an age range of 20–91 years. Seventy-five patients (61.0%) were male and 48 (39.0%) were female. Most isolates (92/123, 74.8%) were obtained from patients in intensive care units. The most common specimen types were urine (n = 47, 38.2%), tracheal aspirate (*n* = 32, 26.0%), and blood/blood-catheter samples (*n* = 30, 24.4%) (Table 1).

### 2.2. Distribution of Carbapenemase Genotypes and Performance of mCIM/eCIM

At least one of the investigated carbapenemase genes was detected in 122 of 123 isolates (99.2%). The most common molecular profile was NDM + OXA-48 co-production (56/123, 45.5%), followed by OXA-48 alone (33/123, 26.8%), KPC alone (23/123, 18.7%), and NDM alone (10/123, 8.1%). One isolate (0.8%) was negative for all five carbapenemase genes investigated. Neither VIM nor IMP was detected in any isolate (Figure 1).

When PCR detection of at least one of the investigated carbapenemase genes was used as the reference, mCIM correctly identified 121 of 122 PCR-positive isolates, corresponding to a sensitivity of 99.2% (95% CI, 95.5–100.0). Because only one isolate was PCR-negative, the assessment of specificity and other diagnostic performance measures was limited (Table 2). Initially, 16 isolates yielded indeterminate mCIM results and were retested. All 16 yielded interpretable results upon repeat testing, and no indeterminate results remained in the final analysis.

Because VIM and IMP were not detected, molecular MBL positivity in this isolate collection corresponded to NDM positivity. Of the 66 NDM-positive isolates identified by PCR, one NDM + OXA-48 co-producer was mCIM-negative and was therefore not included in the eCIM analysis. The performance of eCIM was evaluated among the 121 mCIM-positive isolates, using NDM positivity as the reference. eCIM correctly identified 57 of 65 NDM-positive isolates, corresponding to a sensitivity of 87.7%, specificity of 100%, and overall accuracy of 93.4%. Eight NDM-positive isolates yielded negative eCIM results, and all eight were NDM + OXA-48 co-producers (Table 2). No false-negative eCIM result occurred among isolates carrying NDM alone (0/10), whereas 8 of 55 NDM + OXA-48 co-producing isolates (14.5%) were false negative. This difference was not statistically significant (*p* = 0.339).

### 2.3. Ceftazidime-Avibactam and Colistin Resistance According to Carbapenemase Genotype

Among all isolates, resistance to CZA was detected in 61/123 (49.6%), while 45/123 (36.6%) were resistant to colistin. CZA resistance was observed in 80.0% of isolates carrying NDM alone, 71.4% of NDM + OXA-48 co-producers, 30.4% of isolates carrying KPC alone, and 15.2% of those carrying OXA-48 alone. In the exploratory comparison, the distribution of CZA resistance differed among the four main carbapenemase-positive profiles (*p* < 0.001) (Table 3 and Figure 2). Among NDM + OXA-48-co-producing isolates, CZA resistance was observed in 32 of 40 isolates (80.0%) from Van and 8 of 16 isolates (50.0%) from Kahramanmaraş. Thus, the observed resistance proportion differed between the two study centers.

Colistin resistance was detected in 50.0% of isolates carrying NDM alone, 48.5% of those carrying OXA-48 alone, 37.5% of NDM + OXA-48 co-producers, and 13.0% of isolates carrying KPC alone (Table 3 and Figure 2). Additional analyses of carbapenemase profiles and the distribution of CZA and colistin resistance according to study center are presented in Supplementary Table S2. After accounting for differences between the two study centers, no statistically significant association was observed between carbapenemase profiles and colistin resistance (*p* = 0.117). However, this finding should be interpreted cautiously because of the small number of isolates in certain genotype groups.

In an additional subgroup analysis stratified by specimen type, NDM + OXA-48 co-production was the most frequent carbapenemase profile among urine isolates (27/47, 57.4%), tracheal aspirates (11/32, 34.4%), and blood/blood-catheter isolates (12/30, 40.0%). CZA resistance was observed in 57.4% of urine isolates, 50.0% of tracheal aspirates, 46.7% of blood/blood-catheter isolates, and 28.6% of other specimens. The corresponding colistin resistance rates were 31.9%, 40.6%, 36.7%, and 42.9%, respectively. Exploratory comparisons did not identify statistically significant differences across specimen types in carbapenemase genotype distributions or CZA and colistin resistance rates (Supplementary Table S3).

### 2.4. Associations of NDM/eCIM with CZA Resistance and Colistin MIC

In the exploratory analyses, NDM-positive isolates had higher odds of CZA resistance than NDM-negative isolates (odds ratio [OR], 9.03; 95% confidence interval [CI], 3.97–20.54; *p* < 0.001). After adjustment for study center using logistic regression, NDM positivity remained significantly associated with ceftazidime-avibactam resistance (adjusted OR, 8.92; 95% CI, 3.91–20.35; *p* < 0.001). A center-by-NDM interaction term was also evaluated in the logistic regression model, but the interaction was not statistically significant (likelihood-ratio test, *p* = 0.090). The observed association between eCIM positivity and CZA resistance largely reflects the association between NDM positivity and CZA resistance, rather than an independent finding.

In the exploratory analyses, no statistically significant differences in colistin MIC distributions were detected according to NDM positivity, CZA resistance, or eCIM positivity. The median colistin MIC was 2 mg/L (IQR, 0.5–16) among NDM-positive isolates and 1 mg/L (IQR, 0.5–8) among NDM-negative isolates (*p* = 0.380). The median MIC was 1 mg/L in both CZA-resistant and CZA-susceptible isolates (*p* = 0.963). No statistically significant difference was detected between eCIM-positive and eCIM-negative isolates (*p* = 0.347). Across the four main carbapenemase profiles, the median colistin MIC was 3 mg/L for NDM alone, 2 mg/L for NDM + OXA-48, 2 mg/L for OXA-48 alone, and 0.5 mg/L for KPC alone. The overall comparison did not reach statistical significance (*p* = 0.267) (Table 4). The association between carbapenemase profiles and colistin resistance was also not statistically significant after accounting for study center (Monte Carlo exact test, *p* = 0.117).

## 3. Discussion

Carbapenem-resistant *Klebsiella pneumoniae* is an important cause of healthcare-associated infections, particularly in intensive care units, where treatment options are often limited and different carbapenemases may coexist in the same isolate. In this study, we evaluated the distribution of carbapenemase genes, the agreement of mCIM and eCIM with molecular findings, and the relationship between carbapenemase profiles and CZA and colistin susceptibility in 123 CR-Kp isolates obtained from two centers. At least one of the investigated carbapenemase genes was detected in 99.2% of the isolates, with NDM + OXA-48 co-production being the most common molecular profile (45.5%). mCIM showed high sensitivity for the detection of carbapenemase production, whereas eCIM had lower sensitivity for NDM detection. All eight NDM-positive isolates that were missed by eCIM carried both NDM and OXA-48. In addition, CZA resistance was substantially more frequent among NDM-containing genotypes, whereas no similar association was observed between colistin MIC values and carbapenemase profiles.

Although OXA-48-like carbapenemases have historically been common in Türkiye, recent reports indicate an increasing occurrence of NDM and the coexistence of OXA-48 and NDM. In a multicenter prospective study conducted in 2018–2019, OXA-48-like carbapenemases predominated among CR-Kp causing bloodstream infections, while NDM was detected together with OXA-48-like enzymes in 16% of isolates [15]. More recently, a study of CR-Kp isolates from intensive care units in Istanbul reported NDM + OXA-48 as the most common molecular profile (43%), followed by OXA-48 alone (35%) [16]. Similarly, in a prospective study of CR-Kp bacteremia conducted in Türkiye in 2023–2024, OXA-48 and NDM were detected in 95% and 48.2% of isolates, respectively, and OXA-48 + NDM co-production was identified in 45.7% [17]. In our study, NDM + OXA-48 was also the predominant profile, accounting for 45.5% of isolates, which is consistent with these recent findings from Türkiye.

The coexistence of NDM and OXA-48 has also been increasingly reported outside Türkiye. In a global genomic analysis of CR-Kp isolates carrying multiple carbapenemases, 665 of 832 genomes (79.9%) carrying two carbapenemase genes contained both NDM and an OXA-48-like carbapenemase [18]. Studies from different geographical regions have likewise reported NDM and OXA-48 co-production at varying frequencies [19,20,21,22]. Taken together, these findings indicate that NDM + OXA-48 co-production is not limited to a single geographical setting and represents an important carbapenemase profile across different regions.

Rapid and accurate detection of carbapenemases is important both for guiding antimicrobial therapy and for the timely implementation of infection control measures. Although molecular methods allow direct identification of carbapenemase genes, their routine use may be limited in some laboratories because of cost and infrastructure requirements. Phenotypic methods such as mCIM therefore remain useful in routine microbiology laboratories. In a multicenter study by Pierce et al., mCIM showed high sensitivity, specificity, and interlaboratory reproducibility [23]. Subsequent studies have also reported good performance of mCIM for the phenotypic detection of different carbapenemases [24,25]. In a 2026 study by Güner et al., 96 carbapenem-resistant Enterobacterales isolates were evaluated using real-time PCR as the reference method, and both positive percent agreement and overall percent agreement for mCIM were reported as 97.9%. Two PCR-positive isolates, one producing OXA-48 and the other NDM, yielded negative mCIM results [26]. In our study, mCIM correctly identified 121 of 122 isolates carrying at least one of the investigated carbapenemase genes, corresponding to a sensitivity of 99.2%. These findings support the high sensitivity of mCIM for the phenotypic detection of carbapenemase production. Because only one isolate was PCR-negative, mCIM specificity and negative predictive value were not reported.

eCIM is based on the inhibition of metallo-β-lactamase activity by EDTA and is used to phenotypically identify MBL production in isolates that are positive by mCIM. Because neither VIM nor IMP was detected in our study, molecular MBL positivity corresponded to the presence of NDM. Under these conditions, eCIM showed a sensitivity of 87.7%, specificity of 100%, and overall accuracy of 93.4% for NDM detection. In a study by Sfeir et al., high sensitivity and specificity for distinguishing MBLs from serine carbapenemases were achieved when an appropriate EDTA concentration was used. However, the same study reported a *K. pneumoniae* isolate carrying both NDM and an OXA-48-type carbapenemase that was positive by mCIM but negative by eCIM, and the coexistence of a serine carbapenemase and an MBL was considered an important limitation of the method [24]. Tsai et al. reported a sensitivity of 89.3% and a specificity of 98.7% for MBL detection using the mCIM/eCIM combination [8]. These values are similar to the eCIM performance observed in our study.

An important finding of our study was that all eight NDM-positive isolates with false-negative eCIM results were NDM + OXA-48 co-producers. No false-negative eCIM result was observed among isolates carrying NDM alone, whereas 8 of 55 NDM + OXA-48 co-producing isolates (14.5%) yielded false-negative results. However, the difference between these groups did not reach statistical significance (*p* = 0.339). Therefore, although the occurrence of all false-negative results among NDM + OXA-48 co-producers is noteworthy, our findings alone are insufficient to establish an effect of co-production on eCIM performance. Tsai et al. similarly reported that the presence of an MBL was not correctly identified by the mCIM/eCIM combination in two *E. coli* isolates carrying both OXA-48 and NDM-5 [8]. In a subsequent nine-year molecular epidemiology study from the same group, discordant mCIM/eCIM results were also reported in some isolates co-producing NDM-5 and serine carbapenemases such as OXA-48 or KPC-2 [27]. Similarly, a study comparing different phenotypic carbapenemase detection methods found that isolates producing NDM alone could be detected by eCIM, whereas NDM + OXA-48-like co-producers did not yield positive eCIM results [25].

As NDM/OXA-48 co-production is being reported in different geographical regions and has been associated with hospital outbreaks and regional spread, this limitation may become increasingly relevant for laboratories using eCIM [18,28,29,30,31]. Particularly in settings where NDM and OXA-48 co-production is common, a negative eCIM result should not be considered sufficient to exclude NDM, and molecular confirmation may be appropriate when clinically or epidemiologically indicated.

In our study, the overall rate of CZA resistance was 49.6%, with marked differences according to carbapenemase genotype. CZA resistance was observed in 80.0% of isolates carrying NDM alone and 71.4% of NDM + OXA-48 co-producers, compared with 30.4% of isolates carrying KPC alone and 15.2% of those carrying OXA-48 alone. In our exploratory analysis, NDM-positive isolates had higher odds of CZA resistance (OR, 9.03; 95% CI, 3.97–20.54); however, this association should be interpreted cautiously because no adjustment for multiple comparisons was performed. These findings are broadly consistent with the known activity spectrum of avibactam, which inhibits Ambler class A β-lactamases, including KPC, and certain class D enzymes, including OXA-48, but does not inhibit class B metallo-β-lactamases such as NDM, VIM, and IMP [32]. However, discordant susceptibility results were also observed. Among the 66 NDM-positive isolates, 18 (27.3%) were categorized as susceptible to CZA, whereas resistance was detected in 7 of 23 KPC-only isolates, 5 of 33 OXA-48-only isolates, and the single PCR-negative isolate. Because molecular testing identified carbapenemase gene families without characterizing individual variants, and CZA susceptibility data were available only as categorical results, the mechanisms underlying these discordant findings could not be determined. Findings from Türkiye also support this association. Eren-Kutsoylu et al. reported CZA resistance in 76 of 140 CR-Kp isolates (54.3%). Among isolates that underwent molecular testing, NDM and OXA-48 were detected in 64.5% and 54.8%, respectively, while NDM + OXA-48 co-production was identified in 32.2%. NDM was detected in all CZA-resistant isolates that were molecularly characterized [12]. In an intensive care unit study from Istanbul, 75% of NDM + OXA-48-producing isolates were resistant to CZA, compared with 12% of isolates carrying OXA-48 alone. CZA resistance was also significantly associated with the presence of NDM in that study (OR, 11.67) [16]. These findings are comparable to the genotype-specific distribution of CZA resistance observed in our study. Among NDM + OXA-48 co-producing isolates, the proportion of CZA-resistant isolates differed between Van (80.0%) and Kahramanmaraş (50.0%). Because CZA disk diffusion testing was performed separately at the two centers, inter-laboratory variation may have contributed to this difference.

Although the overall CZA resistance rate in our study was broadly consistent with recent data from Türkiye [12,33], an earlier study from Türkiye that included selected isolates with OXA-48 and KPC activity reported a CZA susceptibility rate of 95.2% [34]. This difference suggests that the distribution of carbapenemase types within the study population should be considered when comparing CZA susceptibility rates across studies.

A similar relationship between carbapenemase type and CZA activity has been reported outside Türkiye. In a 2024 study from India evaluating 89 CR-Kp isolates, CZA resistance was reported in 50.5% of isolates. NDM-1 was detected in most of the CZA-resistant isolates that underwent molecular analysis, while OXA-48 was detected in all of them [35]. In the global INFORM surveillance study, CZA showed high in vitro activity against OXA-48-producing isolates without MBLs [32]. These findings suggest that CZA activity may remain largely preserved in populations dominated by OXA-48 in the absence of MBLs, whereas higher resistance rates may be encountered in isolate populations with a high prevalence of NDM.

It should also be considered that CZA resistance cannot be explained solely by the presence of MBLs. In KPC-producing isolates, variants containing amino acid substitutions in the KPC enzyme may be selected during or after CZA treatment and have been associated with CZA resistance [36,37]. In our study, 30.4% of isolates carrying KPC alone were resistant to CZA. Although KPC variants, porin alterations, efflux systems, and other β-lactamase-mediated mechanisms have been implicated in CZA resistance, these mechanisms were not investigated in the present study. Therefore, the mechanisms underlying CZA resistance in our KPC-positive isolates could not be determined. The observed association between eCIM positivity and CZA resistance largely reflects the association between NDM positivity and CZA resistance, as eCIM serves as a phenotypic indicator of metallo-β-lactamase production. Therefore, the eCIM–CZA association should not be interpreted as an independent finding. eCIM may therefore provide useful preliminary information about the carbapenemase class in laboratories where molecular testing is not immediately available. However, because false-negative eCIM results were observed among NDM + OXA-48 co-producers, CZA susceptibility should not be inferred from the eCIM result alone. The literature on the treatment of CR-Kp infections caused by OXA-48-like and NDM producers similarly indicates that CZA is an important option against OXA-48 producers but is not active alone against NDM-producing isolates [38].

A different pattern was observed for colistin. In our study, the overall colistin resistance rate was 36.6%. A recent intensive care unit study from Istanbul reported a similar rate of 37% [16]. In contrast, a larger Turkish study including 1588 isolates collected between 2023 and 2025 reported a colistin resistance rate of 23% [33]. A 2025 systematic review and meta-analysis of 28 studies including 8916 *K. pneumoniae* isolates from Türkiye between 2004 and 2024 showed an increase in colistin resistance over time and also indicated that differences in susceptibility testing methods and interpretive criteria contributed to the heterogeneity of reported resistance rates [13]. Therefore, comparisons of colistin resistance rates across studies should take into account the testing method, patient population, study period, and the proportion of isolates obtained from high-risk clinical settings such as intensive care units.

The increase in colistin resistance is not limited to Türkiye. A recent global systematic review and meta-analysis of clinical *K. pneumoniae* isolates reported an increase in colistin resistance over time [39]. Another systematic review and meta-analysis focusing on bloodstream infections found a lower overall resistance rate, but higher rates were reported among isolates collected from 2020 onward and among isolates from intensive care patients [40]. In our study, 74.8% of the isolates were obtained from intensive care units, which may partly explain the higher colistin resistance rate compared with some studies based on broader patient populations. However, direct comparisons should be made cautiously because of differences in study populations and specimen types.

Colistin MIC distributions in our study were not significantly associated with NDM positivity, CZA resistance, or eCIM positivity. Similarly, no significant difference in quantitative colistin MIC values was found among the four main carbapenemase profiles. In this exploratory analysis, no statistically significant association was detected between carbapenemase genotype and colistin MIC values; however, this finding does not exclude a potential relationship. The molecular basis of colistin resistance differs from that of carbapenem resistance, and alterations in chromosomal regulatory systems can play an important role in *K. pneumoniae* [41]. A genomic study from Türkiye published in 2026 investigated carbapenem- and colistin-resistant *K. pneumoniae* isolates and showed that resistance to these two antimicrobial classes may coexist through the accumulation of different resistance mechanisms within the same isolates [42]. Because the molecular mechanisms responsible for colistin resistance were not investigated in our study, however, the lack of an association between carbapenemase profiles and colistin MIC values cannot be explained mechanistically from our data.

The methodology used for susceptibility testing is also important when interpreting colistin results. Because of the diffusion characteristics of polymyxins in agar and the performance limitations of some susceptibility testing methods, reliable determination of colistin MICs is particularly important. The international consensus guidelines for polymyxins recognize broth microdilution as the reference method for colistin susceptibility testing [43]. The use of broth microdilution to determine colistin MICs in our study therefore represents a methodological strength of the phenotypic colistin assessment.

Taken together, our findings showed different patterns of association between carbapenemase genotypes and susceptibility to CZA and colistin. NDM positivity was associated with higher odds of CZA resistance, whereas no statistically significant differences in colistin MIC distributions were detected according to carbapenemase genotype. These associations should be interpreted cautiously because no adjustment for multiple comparisons was performed. Although established antimicrobial resistance mechanisms provide relevant biological context, the molecular mechanisms underlying the observed susceptibility patterns were not investigated in the present study [32,38,39,41].

This study has several limitations. The study was conducted at only two hospitals in Türkiye, which limits the geographical representativeness of the isolate collection and the generalizability of the findings to other healthcare settings. Molecular testing was limited to KPC, NDM, VIM, IMP, and OXA-48; therefore, the presence of another carbapenemase or a non-carbapenemase resistance mechanism cannot be excluded in the single isolate that was negative for all five investigated genes. NDM, OXA-48, and KPC variants were not characterized, and clonal relatedness and whole-genome sequences of the isolates were not investigated. A separate carbapenemase-positive quality control strain was not included in the phenotypic assays, which should be considered when interpreting the diagnostic performance findings. Ceftazidime-avibactam susceptibility data were available only as categorical results (susceptible or resistant) from routine laboratory records. The absence of individual inhibition zone diameters and MIC values limited further evaluation of discordant genotype–susceptibility findings. The relatively small number of isolates carrying NDM alone (*n* = 10) also limited comparisons of eCIM performance across carbapenemase profiles. Although all eight false-negative eCIM results occurred among NDM + OXA-48 co-producing isolates (8/55), compared with none among isolates carrying NDM alone (0/10), this difference was not statistically significant (*p* = 0.339) and does not establish an effect of carbapenemase co-production on eCIM performance. Most isolates were obtained from intensive care units (74.8%), resulting in an ICU-enriched study population and potential selection bias. Therefore, the observed carbapenemase distributions and antimicrobial resistance rates should not be extrapolated to non-ICU patient populations. The molecular mechanisms responsible for colistin resistance were not investigated, limiting the mechanistic interpretation of the colistin findings. In addition, possible explanations for some of the differences observed between the two centers, including patient characteristics, local antimicrobial use, or clonal spread, could not be evaluated within the scope of this study. Finally, multiple comparisons were performed when assessing the relationships between carbapenemase genotypes and ceftazidime-avibactam and colistin susceptibility, without statistical adjustment for multiple testing. These findings should therefore be considered hypothesis-generating rather than confirmatory.

Despite these limitations, this study has several strengths. Isolates were obtained from two different centers, only the first isolate from each patient was included, and phenotypic and molecular carbapenemase results were directly compared in the same isolates. CZA and colistin susceptibility results were also evaluated together with carbapenemase profiles. In addition, evaluating mCIM/eCIM performance in relation to molecular carbapenemase profiles allowed us to describe the distribution of false-negative eCIM results among isolates with different carbapenemase genotypes, without establishing a causal relationship between enzyme co-production and test performance.

## 4. Materials and Methods

### 4.1. Study Design and Isolate Selection

This observational laboratory study was conducted in the microbiology laboratories of Kahramanmaraş Necip Fazıl City Hospital and University of Health Sciences Van Training and Research Hospital. A total of 123 CR-Kp isolates recovered from various clinical specimens of patients aged ≥18 years during routine diagnostic microbiology procedures between March and September 2026 were included. When more than one isolate was recovered from the same patient, only the first isolate was included, and subsequent isolates were excluded. The isolates were obtained as part of routine diagnostic laboratory procedures and stored at −80 °C until the phenotypic and molecular analyses were performed. Molecular detection of carbapenemase genes was performed at Van Training and Research Hospital, whereas phenotypic carbapenemase testing (mCIM and eCIM) was performed at Kahramanmaraş Necip Fazıl City Hospital. Routine antimicrobial susceptibility testing was performed at each participating hospital, whereas colistin broth microdilution testing was performed centrally at Kahramanmaraş Necip Fazıl City Hospital.

### 4.2. Bacterial Identification and Antimicrobial Susceptibility Testing

The isolates were identified using colony morphology, Gram staining, conventional biochemical tests, and the VITEK^®^ 2 Compact system (bioMérieux, Marcy-l’Étoile, France). Species-level identification was performed according to the manufacturer’s instructions.

Routine antimicrobial susceptibility testing and determination of minimum inhibitory concentration (MIC) values for carbapenems were performed using the VITEK^®^ 2 Compact system with AST-N322 cards (bioMérieux, Marcy-l’Étoile, France). Carbapenem susceptibility was interpreted according to the 2026 clinical breakpoints of the European Committee on Antimicrobial Susceptibility Testing (EUCAST), and isolates classified as carbapenem-resistant were included in the study [44].

CZA susceptibility was determined by the disk diffusion method. Bacterial suspensions adjusted to a turbidity of 0.5 McFarland were evenly inoculated onto Mueller–Hinton agar, and 10/4 µg ceftazidime-avibactam disks (Bioanalyse, Ankara, Türkiye) were placed on the agar surface. Plates were incubated at 35± 2 °C for 16–20 h. After incubation, susceptibility results were interpreted according to the EUCAST 2026 clinical breakpoints [44]. For the present study, CZA susceptibility data were obtained retrospectively from routine laboratory records and were available only as categorical results (susceptible or resistant). Individual inhibition zone diameters were not retained in the study dataset.

Colistin MICs were determined using the broth microdilution (BMD) method. An antibiotic solution prepared from colistin sulfate powder (Sigma-Aldrich, St. Louis, MO, USA) was serially two-fold diluted in cation-adjusted Mueller–Hinton broth in 96-well microtiter plates. The tested colistin concentration range was 0.25–128 mg/L. Each plate included a growth-control well without antibiotic and a sterility-control well without bacterial inoculum. Plates were incubated at 35 ± 2 °C for 16–20 h. The MIC was defined as the lowest colistin concentration with no visible bacterial growth. Results were interpreted according to the EUCAST 2026 clinical breakpoints [44]. Colistin MIC testing was performed for all isolates at Kahramanmaraş Necip Fazıl City Hospital using the same broth microdilution protocol, regardless of the originating study center.

*Escherichia coli* ATCC 25922 was used as the quality-control strain for antimicrobial susceptibility testing. The quality-control MIC results for colistin were within the accepted range.

### 4.3. Phenotypic Detection of Carbapenemase Production

The mCIM was used for the phenotypic detection of carbapenemase production, while the eCIM was used to investigate MBL production. Both tests were performed according to the recommendations of the Clinical and Laboratory Standards Institute (CLSI) M100 [45]. Isolates yielding indeterminate mCIM results during initial testing were retested using the same procedure, and the results obtained after repeat testing were used in the final analysis.

For mCIM, bacteria were taken from an 18–24 h pure culture using a sterile 1 µL loop and suspended homogeneously in a sterile tube containing 2 mL of tryptic soy broth (TSB). A 10 µg meropenem disk (Bioanalyse, Türkiye) was placed into the suspension, ensuring that the disk remained completely immersed. The tubes were incubated at 35 ± 2 °C for 4 h ± 15 min without agitation. Near the end of this incubation period, a 0.5 McFarland suspension of the indicator strain, *Escherichia coli* ATCC 25922, was prepared and evenly inoculated onto the surface of Mueller–Hinton agar (MHA) using a sterile swab. After the 4 h incubation, the meropenem disk was removed from the TSB using sterile forceps and placed on the MHA plate inoculated with *E. coli* ATCC 25922. Plates were incubated at 35 ± 2 °C for 18–24 h.

After incubation, the inhibition zone diameter around the meropenem disk was measured and interpreted according to CLSI criteria. An inhibition zone of 6–15 mm, or a zone of 16–18 mm with pinpoint colonies within the inhibition zone, was interpreted as positive for carbapenemase production. A zone diameter of ≥19 mm was interpreted as negative. A result was considered indeterminate when pinpoint colonies were present within a zone of ≥19 mm or when no colonies were observed within a 16–18 mm inhibition zone (Figure 3).

For eCIM, the test was performed only for isolates with a positive mCIM result. The procedure was carried out in parallel with mCIM, with the addition of 20 µL of 0.5 M EDTA to 2 mL of TSB before the meropenem disk was added. The remaining incubation and inoculation steps were performed as described for mCIM. After incubation, the inhibition zone diameter obtained with eCIM was compared with that obtained with mCIM. An increase of ≥5 mm in the eCIM inhibition zone compared with the mCIM zone was interpreted as positive for MBL production, whereas an increase of ≤4 mm was interpreted as negative. eCIM results were not interpreted for isolates with a negative mCIM result (Figure 3).

### 4.4. Molecular Detection of Carbapenemase Genes

Before molecular analysis, isolates stored at −80 °C were subcultured onto 5% sheep blood agar and incubated at 35 ± 2 °C for 24 h. DNA extraction was performed from fresh bacterial cultures using the Bio-Speedy^®^ Rapid Nucleic Acid Extraction Kit (Bioeksen, İstanbul, Türkiye) and the Zybio EXM3000 extraction system according to the manufacturers’ instructions.

Carbapenemase genes were investigated using the Bio-Speedy^®^ Carbapenem Resistance PCR Kit (Bioeksen, Türkiye). Multiplex real-time PCR was performed using the Mic qPCR System (Bio Molecular Systems, Upper Coomera, QLD, Australia). The assay targeted the KPC, NDM, VIM, IMP, and OXA-48 carbapenemase genes. Positive, negative, and internal controls supplied with the kit were included in each run, and results were interpreted according to the manufacturer’s instructions.

Molecular carbapenemase profiles were defined according to the detected gene combinations. Isolates carrying two or more of the investigated carbapenemase genes were classified as carrying multiple carbapenemase genes.

### 4.5. Statistical Analysis

Statistical analyses were performed using IBM SPSS Statistics version 26.0 (IBM Corp., Armonk, NY, USA). The distribution of continuous variables was assessed using the Shapiro–Wilk test. Continuous variables that were not normally distributed were summarized as the median and interquartile range (IQR), while categorical variables were presented as the number and percentage.

Categorical variables were compared using Pearson’s chi-square test or Fisher’s exact test, as appropriate. Comparisons of continuous variables between two independent groups were performed using the Mann–Whitney U test, while comparisons among more than two groups were performed using the Kruskal–Wallis test.

PCR was used as the reference method for evaluating the diagnostic performance of mCIM and eCIM. For mCIM, the presence of at least one of the investigated carbapenemase genes (KPC, NDM, VIM, IMP, or OXA-48) was considered molecularly positive. For the evaluation of eCIM, molecular MBL positivity was defined as the presence of NDM, VIM, or IMP. For mCIM, only sensitivity was reported because only one isolate was PCR-negative, precluding reliable estimation of specificity and negative predictive value. For eCIM, sensitivity, specificity, positive predictive value, negative predictive value, and overall accuracy were evaluated using PCR-based results as the reference.

The distribution of CZA and colistin resistance was compared according to carbapenemase genotype. The association between NDM positivity and CZA resistance was evaluated using an odds ratio (OR) with a 95% confidence interval (CI). Colistin MIC distributions were compared according to NDM positivity, CZA resistance, eCIM positivity, and the major carbapenemase profiles using non-parametric tests, as appropriate. The association between NDM positivity and ceftazidime-avibactam resistance was additionally evaluated using logistic regression adjusted for study center. A center-by-NDM interaction term was included in a separate logistic regression model and evaluated using a likelihood-ratio test. The association between carbapenemase profiles and colistin resistance was evaluated using a center-stratified Monte Carlo exact test with 100,000 simulations.

Additional exploratory subgroup analyses were performed according to specimen type. Categorical variables were compared using appropriate contingency-table methods, with exact or simulation-based approaches considered when expected cell counts were small. No adjustment for multiple comparisons was performed. All statistical tests were two-sided, and *p* < 0.05 was considered statistically significant.

## 5. Conclusions

In this study, NDM + OXA-48 co-production was the most common carbapenemase profile among CR-Kp isolates. mCIM showed high sensitivity for the phenotypic detection of carbapenemase production, whereas eCIM had lower sensitivity for NDM detection. All eight false-negative eCIM results occurred among NDM + OXA-48 co-producing isolates (8/55), compared with none among isolates carrying NDM alone (0/10); however, this difference was not statistically significant (*p* = 0.339). NDM-positive isolates had higher odds of CZA resistance, while no statistically significant differences in colistin MIC distributions were detected among carbapenemase profiles. These associations should be interpreted cautiously because multiple comparisons were performed without statistical adjustment. Overall, our findings highlight the potential value of interpreting phenotypic carbapenemase test results alongside molecular carbapenemase profiles and antimicrobial susceptibility data, particularly in settings where NDM and OXA-48 co-production is common.

## Figures and Tables

**Figure 1:**
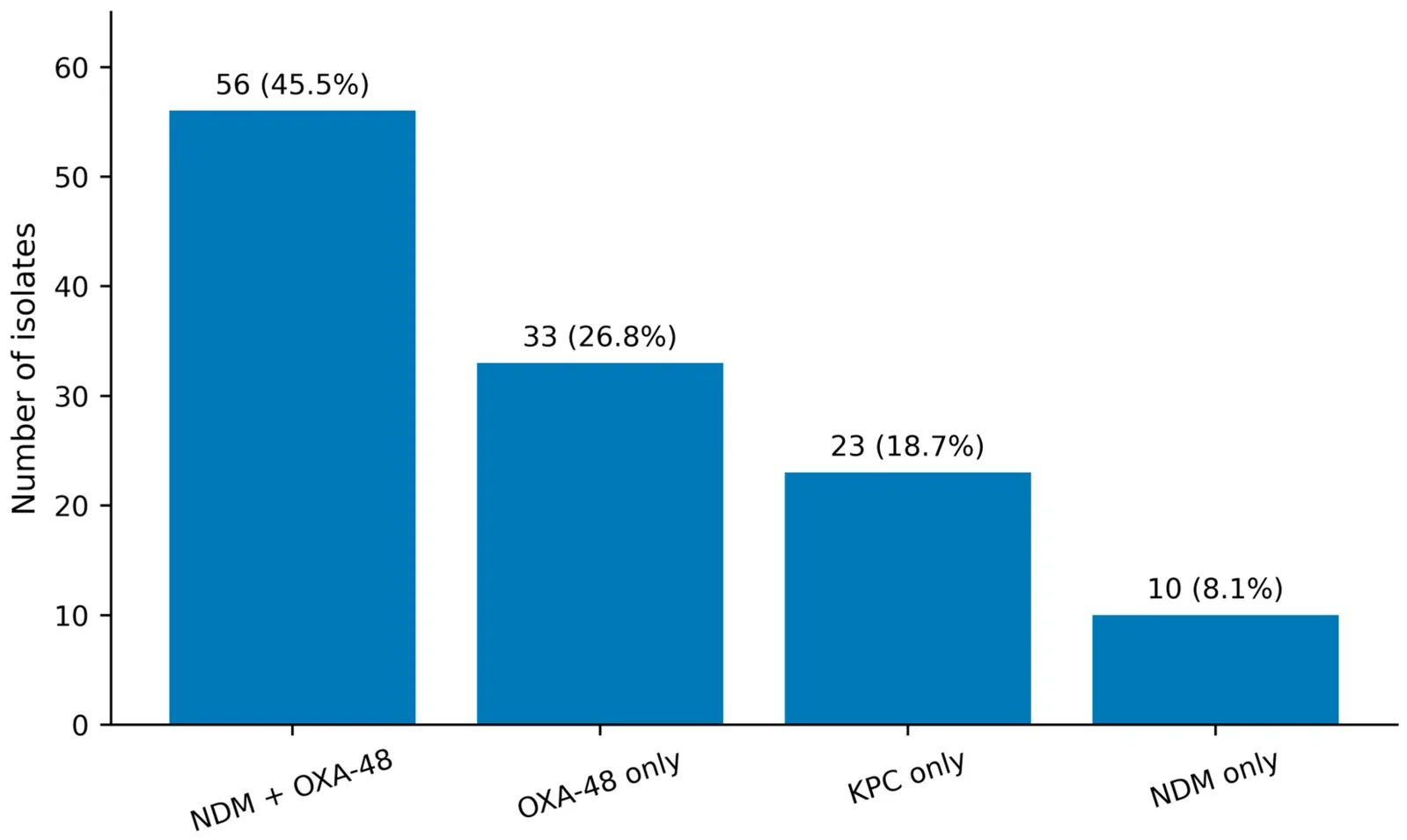
Distribution of PCR-defined carbapenemase profiles among carbapenem-resistant *Klebsiella pneumoniae* isolates. The single isolate negative for all five investigated carbapenemase genes (0.8%) is not displayed.

**Figure 2:**
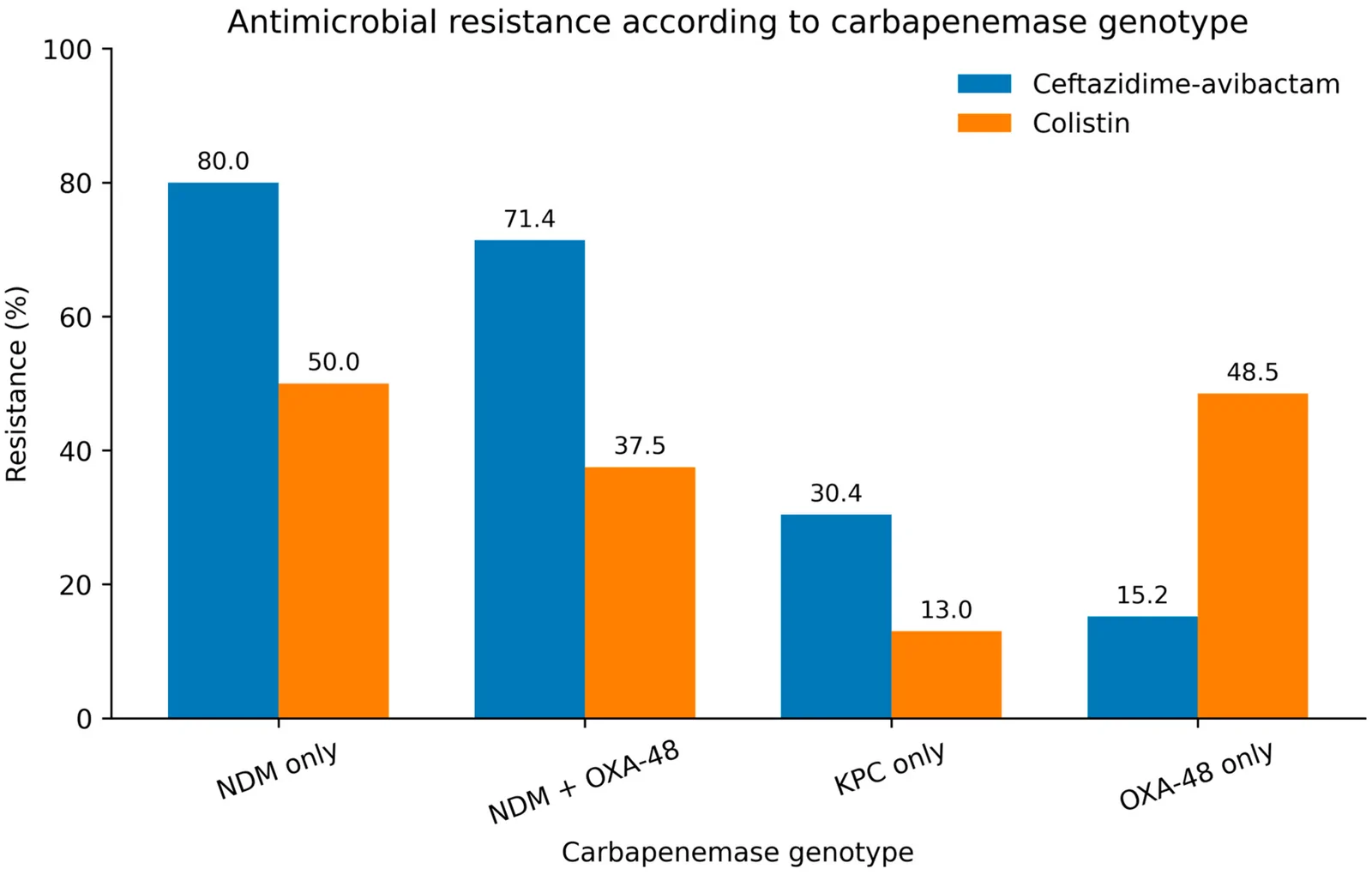
Ceftazidime-avibactam and colistin resistance rates according to PCR-defined carbapenemase genotypes. The single isolate negative for the investigated carbapenemase genes by PCR was excluded from the molecular subgroup comparison.

**Figure 3:**
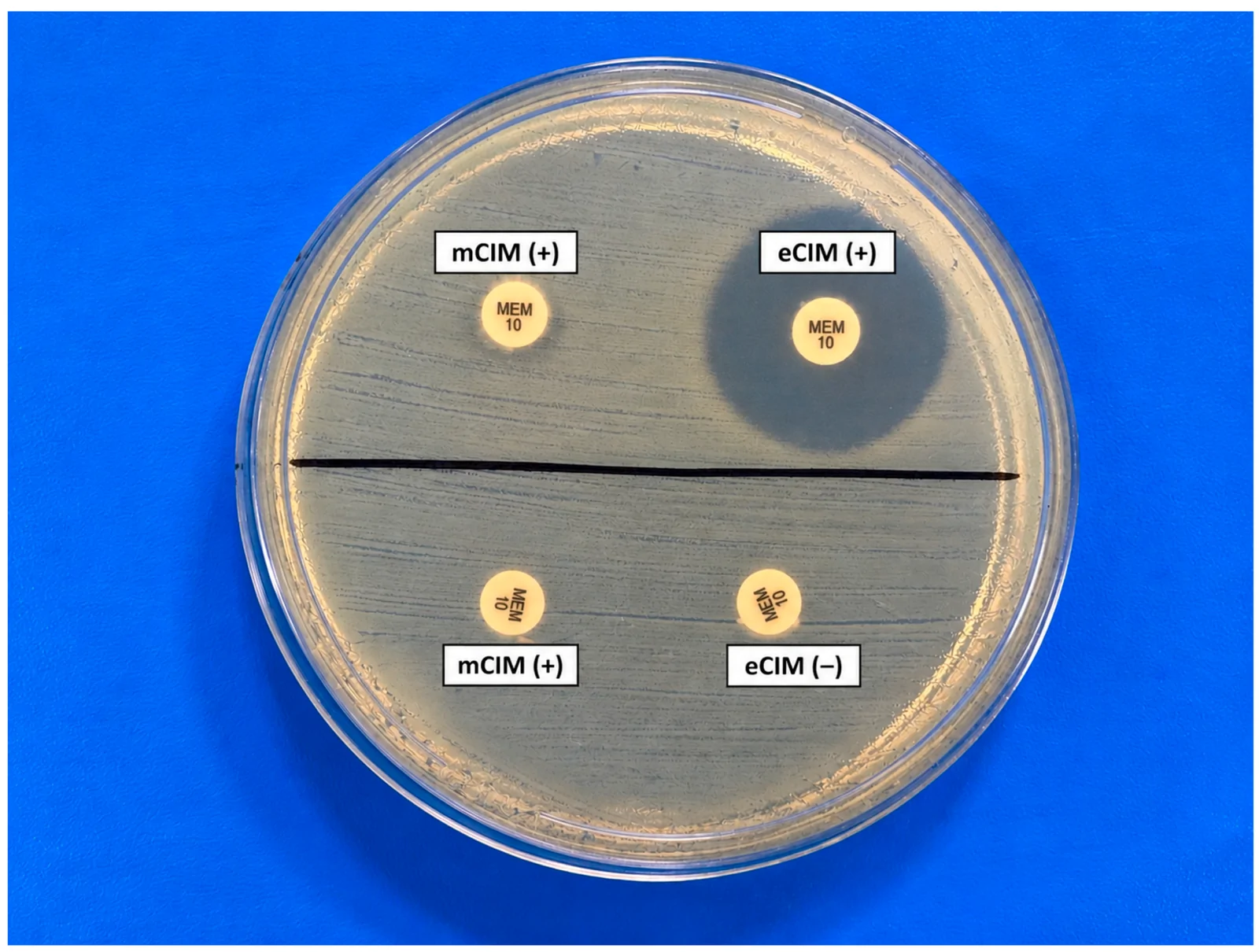
Representative mCIM and eCIM results from two different isolates. The upper half of the plate shows positive mCIM and eCIM results, indicating phenotypic carbapenemase and metallo-β-lactamase activity. The lower half shows a positive mCIM result and a negative eCIM result, indicating carbapenemase activity without detectable metallo-β-lactamase activity by eCIM.

**Table 1:** Clinical and microbiological characteristics of the study population.

Characteristic	*n* (%)/Median (IQR)
Total isolates	123
Age, years, median (IQR)	70 (57.5–78.0)
Sex, male/female	75 (61.0)/48 (39.0)
Intensive care unit	92 (74.8)
Study center	
University of Health Sciences Van Training and Research Hospital	72 (58.5)
Kahramanmaraş Necip Fazıl City Hospital	51 (41.5)
Specimen type	
Urine	47 (38.2)
Tracheal aspirate	32 (26.0)
Blood/blood-catheter	30 (24.4)
Other	14 (11.4)

Note: Data are presented as *n* (%) unless otherwise indicated. IQR, interquartile range.

**Table 2:** Diagnostic performance of mCIM and eCIM using PCR-based reference definitions.

Test	TP/FN/FP/TN	Sensitivity, % (95% CI)	Specificity, % (95% CI)	PPV, %	NPV, %	Accuracy, %	κ
mCIM vs. any carbapenemase gene	121/1/0/1	99.2 (95.5–100.0)	-	-	-	-	-
eCIM vs. MBL/NDM ^†^	57/8/0/56	87.7 (77.2–94.5)	100.0 (93.6–100.0)	100.0	87.5	93.4	0.868

Note: PCR-defined carbapenemase positivity was defined as the detection of at least one of the KPC, NDM, VIM, IMP, or OXA-48 genes. Because VIM and IMP were not detected, molecular MBL positivity corresponded to NDM positivity. ^†^ eCIM performance was evaluated among mCIM-positive isolates (*n* = 121). All eight false-negative eCIM results occurred in NDM + OXA-48 co-producing isolates. For mCIM, only sensitivity is reported as a diagnostic performance measure because only one isolate was PCR-negative. Dashes indicate measures not reported.

**Table 3:** Distribution of ceftazidim-avibaktam and colistin resistance among carbapenemase genotype groups.

Carbapenemase Profile	Total, *n*	CZA-Resistant, *n* (%)	Colistin-Resistant, *n* (%)
NDM only	10	8 (80.0)	5 (50.0)
NDM + OXA-48	56	40 (71.4)	21 (37.5)
KPC only	23	7 (30.4)	3 (13.0)
OXA-48 only	33	5 (15.2)	16 (48.5)
PCR carbapenemase-negative *	1	1 (100.0)	0 (0.0)
Total	123	61 (49.6)	45 (36.6)

Note: An exploratory comparison showed differences in CZA resistance among the four main carbapenemase-positive profiles (*p* < 0.001; unadjusted for multiple comparisons). * The single PCR carbapenemase-negative isolate is shown for completeness and should not be interpreted as a stable molecular subgroup. CZA, ceftazidime-avibactam.

**Table 4:** Main associations of molecular and phenotypic characteristics with ceftazidime-avibactam resistance and colistin MIC.

Analysis	Effect/Distribution	95% CI or IQR	*p*-Value
NDM positivity and CZA resistance	OR 9.03	95% CI 3.97–20.54	<0.001
Colistin MIC: NDM+ vs. NDM−	2 vs. 1 mg/L	IQR 0.5–16 vs. 0.5–8	0.380
Colistin MIC: CZA-R vs. CZA-S	1 vs. 1 mg/L	IQR 0.5–8 vs. 0.5–14	0.963
Colistin MIC: eCIM+ vs. eCIM− ^†^	2 vs. 1 mg/L	IQR 0.25–16 vs. 0.5–8	0.347
Colistin MIC across the four PCR-defined profiles ^‡^	NDM only 3; NDM+OXA-48 2; OXA-48 only 2; KPC only 0.5 mg/L	Median values	0.267

Note: OR, odds ratio; CI, confidence interval; MIC, minimum inhibitory concentration; IQR, interquartile range. Colistin MIC values reported as ≤0.25 mg/L and ≥128 mg/L were assigned values of 0.25 and 128 mg/L, respectively, for quantitative analyses. ^†^ Analyses involving eCIM were performed among mCIM-positive isolates (*n* = 121). ^‡^ Comparison included the four main PCR-defined carbapenemase-positive profiles: NDM only, NDM + OXA-48, OXA-48 only, and KPC only.

## Data Availability

The data are not publicly available due to privacy and ethical restrictions but are available from the corresponding author upon reasonable request.

## Supplementary Materials

The following supporting information can be downloaded at: https://www.mdpi.com/article/10.3390/ijms27209307/s1.

## Abbreviations

BMD	Broth microdilution
CAMHB	Cation-adjusted Mueller–Hinton broth
CI	Confidence interval
CLSI	Clinical and Laboratory Standards Institute
CR-Kp	Carbapenem-resistant *Klebsiella pneumoniae*
CZA	Ceftazidime-avibactam
eCIM	EDTA-modified carbapenem inactivation method
EUCAST	European Committee on Antimicrobial Susceptibility Testing
FN	False negative
FP	False positive
IMP	Imipenemase
IQR	Interquartile range
KPC	*Klebsiella pneumoniae* carbapenemase
MBL	Metallo-β-lactamase
mCIM	Modified carbapenem inactivation method
MHA	Mueller–Hinton agar
MIC	Minimum inhibitory concentration
NDM	New Delhi metallo-β-lactamase
NPV	Negative predictive value
OR	Odds ratio
OXA-48	Oxacillinase-48
PCR	Polymerase chain reaction
PPV	Positive predictive value
TN	True negative
TP	True positive
TSB	Tryptic soy broth
VIM	Verona integron-encoded metallo-β-lactamase
